# Chronic nature exposure does not moderate affective and attentional effects of acute nature exposure

**DOI:** 10.3389/fpsyg.2026.1731261

**Published:** 2026-02-12

**Authors:** Janet Trammell, Minjoo Kim, Hangrui Tian

**Affiliations:** Social Science Division, Seaver College, Pepperdine University, Malibu, CA, United States

**Keywords:** affect, attention, environment, exposure, nature, restoration

## Abstract

Research consistently demonstrates that exposure to natural environments benefits affect and attention, though findings remain mixed, with some studies reporting negligible or opposite effects. One proposed explanation is that chronic nature exposure may attenuate the benefits of acute exposure. The current study tested whether chronic high (residing in a nature utopia) or low (residing in a nature-deficient environment) nature exposure moderates the effects of an acute nature exposure on affect and cognition. A total of 456 U.S. adults were randomly assigned to view a 5-min video of a natural, urban, or geometric (control) environment. Affect (positive and negative affect, stress, happiness), and attention (digit span backwards) were assessed pre- and post-exposure and perceived restorativeness was assessed post-exposure. As hypothesized, the nature environment was perceived as the most restorative, and both the nature and control conditions reduced negative affect more than the urban condition, but did not differ from each other, supporting the Nature-as-Reward hypothesis. These findings highlight the importance of considering stimulus pleasantness more broadly, as beneficial outcomes may stem from reward responses. No effects of the environment on positive affect, happiness, or attention were found. Moderation analyses conducted with a subsample (*N* = 357) of those living in either the low or high extremes of chronic nature exposure revealed that chronic exposure did not moderate the relationship between acute exposure condition and the outcome measures. These findings suggest that while nature is restorative, its affective and cognitive benefits do not depend on prior chronic exposure.

## Introduction

As urbanization continues to expand, spending time in nature has become a scarce experience for some individuals. However, numerous studies have demonstrated the positive benefits of having contact with nature as opposed to contact with urban environments. While the majority of published research has shown various positive effects of exposure to nature, the findings in this area are not always consistent, prompting a deeper exploration into why some studies report weaker, non-existent, or even opposite effects than others.

One of the most robust effects of exposure to nature is improved psychological wellbeing after exposure, including improvements in mood (Berman et al., 2012; Nisbet and Zelenski, 2011) and decreased levels of depression, stress, and anxiety (Bratman et al., 2015; Browning et al., 2024; de Kort et al., 2006; Jimenez et al., 2021; Liu et al., 2023). In addition to acute exposure, chronic nature exposure (e.g., residing in greener areas or in close proximity to nature) is associated with better wellbeing, positive affect, and mood, and reduced levels of depression, anxiety, and stress (Beyer et al., 2014, 2016; Cox et al., 2017; Garrett et al., 2021; Li et al., 2018; Roe et al., 2013; Sato and Conner, 2013; Thompson et al., 2012).

Similar to positive mental health benefits, acute exposure to nature also benefits individuals' cognitive functions, primarily in the areas of working memory and attentional performance (Berman et al., 2008, 2012; Berto, 2005; Bratman et al., 2015; Hartig et al., 2003; Jimenez et al., 2021; Ohly et al., 2016; Stevenson et al., 2018; Trammell and Aguilar, 2021; Van Hedger et al., 2019). As with affective measures, residing near green spaces is beneficial for cognition in both children (Dadvand et al., 2015) and adults (Zijlema et al., 2017).

Several theories account for beneficial effects of nature on affect and cognition. The Stress Reduction Theory (SRT; Ulrich, 1981, 1983; Ulrich et al., 1991) proposed that nature evokes evolutionary responses of safety that foster positive emotions and reduce stress, evident by lower physiological stress indicators (e.g., heart rate, cortisol). The Attention Restoration Theory (ART; Kaplan and Kaplan, 1989; Kaplan, 1995; Kaplan and Berman, 2010) posits that attentional resources may be restored and recovered by exposure to natural environments through specific qualities of nature that foster “soft fascination,” or undemanding attentional engagement, which is in direct contrast to the more focused attentional demands of non-natural environments. ART has been investigated extensively, and results have generally supported the theory, particularly for working memory and attentional control tasks, and when cognitive resources have been fatigued (see Bell et al., 2025, for a meta-analysis). However, while ART remains the dominant theory for cognitive effects of nature, it has been criticized for vague operationalisms and limited support for the assumption of resource recovery (Joye and Dewitte, 2018; Joye et al., 2022).

More recently, Joye et al. (2024b,a) have proposed the nature-as-reward (NAR) hypothesis, part of goal-discrepancy theory, that may account for some inconsistent results. Particularly, instead of resource depletion and restoration as proposed in ART, exposure to nature is beneficial for cognitive tasks because of the pleasurable aspects of nature, which act as a reward promoting cognitive improvement. In contrast, exposure to other environments (e.g., urban) is not rewarding, which results in task disengagement and decreased performance. Thus, only nature that is rewarding (i.e., beautiful), should result in cognitive benefits. In support of this hypothesis, pleasant fractals and beautiful nature result in greater effort and motivation than less beautiful fractals or nature (Joye et al., 2024b).

However, although numerous studies support ART and N-A-R theories through affective benefits of nature exposure, findings are not always consistent. For instance, indoor exercise has been found to benefit affect more than outdoor nature exercise (Trammell and Aguilar, 2021), and some studies report no differences between nature and urban walks (Jones et al., 2021). Others even suggest negative associations, such as increased green space exposure associated with higher stress during commutes (Liu et al., 2024). Further, a meta-analysis (Roberts et al., 2019) found only small effects on depressive mood and highlighted concerns about study quality and bias.

Likewise, inconsistencies exist in the effects of nature exposure on cognition. Several studies and meta-analyses report no improvements in working memory, executive attention, recall, recognition memory, and additional effects including vigilance, impulse control, and processing speed (Neilson et al., 2021; Scott et al., 2023; Trammell and Aguilar, 2021; Stevenson et al., 2018; Trammell et al., 2024). These findings suggest that benefits of nature exposure may be confined to specific cognitive tasks, such as those requiring moderate rather than intense attention (Trammell and Aguilar, 2021). Indeed, a recent meta-analysis (Bell et al., 2025) showed strongest effects for attentional control and working memory, but negligible effects for other cognitive processes (Bell et al., 2025).

One proposed explanation of the inconsistent effects of natural space is adaptation, suggested by Trammell and Aguilar (2021). This study found that participants in Malibu, California–an environment with abundant natural beauty–did not experience the predicted effects on affect and attention from an acute nature exposure, possibly due to repeated chronic exposure to nature of these participants. This is known as hedonic adaptation (Frederick and Loewenstein, 1999), wherein the response to stimuli–in this case, nature–decreases over time as individuals adapt to their circumstances. The affective adaptation model (Wilson and Gilbert, 2008) suggests that affective response diminishes over time as attention to stimuli declines following repeated exposure. Indeed, Trammell and Aguilar (2021) found that natural settings increased affect less than indoor ones, with participants reporting only somewhat, rather than strongly or moderately, attending to the natural environment. Similarly, Trammell et al. (2024) found small affective but not cognitive benefits of nature walks among students attending a University in Malibu, further suggesting frequent exposure to nature may weaken nature's acute effects. In support of this idea, White et al. (2019) showed improved wellbeing as exposure to nature increased up to 120 weekly minutes, but benefits start diminishing after 120 weekly minutes. However, the lack of residential data in these studies (Trammell and Aguilar, 2021; Trammell et al., 2024) prevents firm conclusions about adaptation.

While the concept of adaptation as a result of chronic nature exposure may account for weakened or lack of positive effects of an acute nature exposure, it cannot explain why increased chronic exposure to nature (such as residing in areas with higher percentages of or in close proximity to green space) is associated with better wellbeing, greater positive affect and mood, and reduced levels of depression, anxiety, and stress (Beyer et al., 2014; Garrett et al., 2021; Li et al., 2018; Thompson et al., 2012). In fact, research also shows sustained mental health improvements after moving to greener areas (Alcock et al., 2014). When considered together, it may be that chronic nature exposure results in long-term benefits to wellbeing, but may weaken the acute effects *of each individual exposure*. As this possibility remains underexplored, the current study aims to assess whether acute exposure differs in its effects for individuals with chronic vs. minimal prior exposure, such that acute nature exposure may result in weaker affective and cognitive benefits for those with chronic nature exposure compared to those without chronic exposure. This study is the first, to our knowledge, to investigate how chronic exposure to nature may moderate the effects of an acute exposure.

Additionally, while it is impossible to retroactively determine if some of the past inconsistencies in prior research can be attributed to participants finding urban and natural settings equally rewarding, the N-A-R hypothesis may account for some of this variability. Thus, we have included a control condition of geometric shapes, as fractals and geometric shapes of varying complexity are often rated as beautiful/pleasant (Bies et al., 2016; Gómez-Puerto et al., 2016; Lavdas and Schirpke, 2020; Redies et al., 2007). If the N-A-R hypothesis is supported, both the nature and geometric conditions should yield similar effects: a larger improvement in affect and attention than the urban condition that contains no natural or pleasant geometric elements.

In line with the predominant ART theory and past research, we first hypothesize that compared to exposure to urban or control environments, a brief exposure to a nature environment will result in a larger decrease in stress and negative affect (NA), a larger increase in positive affect (PA) and happiness, better performance on a measure of attention (digit span backwards task), and greater perceived restorativeness. If instead, the nature condition does not differ from the geometric shapes control condition, results would support the N-A-R hypothesis. Second, for the variable of adaptation, conceptualized as chronic (residential) exposure to nature-rich environments, we hypothesize that chronic exposure will moderate the relationship between environment (nature, urban, or control) and the outcome variables of affect (stress, NA, PA, happiness) and attention. Specifically, exposure is not expected to predict differences in the outcome measures for those in the control or urban environments, but those in the Nature environment with chronic nature exposure (e.g., residing in a “Nature Utopia”) will show a smaller decrease in stress and NA, a smaller increase in PA and happiness, weaker performance on the DSB, and less perceived restorativeness than those in the Nature environment with minimal exposure to nature (e.g., residing in “Nature Deficient” areas).

## Method

This research was approved by and adhered to all ethical processes of the Institutional Review Board of Pepperdine University. The data were collected over 10 months, from September 2024 to May 2025. This study was part of a larger data collection; the method, hypotheses, and analyses relevant to this study were pre-registered at https://osf.io/uawh7/?view_only=819d80f108734625ad96c59d6c22857f.

### Participants

Adult participants were recruited via a convenience sample from the online survey platform Prolific. A power analysis using the program G^*^Power 3.1 indicated a sample size of 244 participants was needed to detect a small to medium effect (η_*p*_^2^ = 0.03) at an alpha level of 0.05. To account for potential missing data or failure to complete the experiment, we initially planned to recruit an additional 12.5% for a total of 275 participants. After reaching 275 participants and calculating a residential nature exposure score (see Nature Exposure section below) for each participant, it was apparent that the majority of participants in the sample had very high exposure (“Nature Utopia” or “Nature Rich”). Thus, to obtain a minimum of 30% of the sample residing in a “Nature Deficient” environment, we recruited an additional 219 participants by primarily targeting zip codes with Nature Deficient scores. This resulted in a final sample of 494 U.S. adults, of which 38 participants (7.69%) were removed due to failing an attention check (*N* = 5), declining to consent to participate (*N* = 2), or not completing at least 90% of dependent measures (*N* = 31), resulting in a final overall sample of 456 and a subsample of 357 (limited to those with either the lowest, Nature Deficient, or highest, Nature Utopia, residential nature exposure). Participants received payment at the Prolific-recommended rate of $12 per hour for their participation in the study.

To account for the possibility that SES may be conflated with chronic nature exposure, we conducted an independent-means *t*-test on SES with the factor of Exposure: Nature Deficient (*M* = 2.02, *SD* = 0.85) vs. Nature Utopia (*M* = 1.89, *SD* = 0.79). There were no differences in SES between groups, *t*_(355)_ = 1.69, *p* = 0.09, *d* = 0.18. See Table 1 for participant demographics, including gender, race and ethnicity, SES, age, and the amount and quality (Nature Score and Leaf Rating) of residential nature exposure. All 456 participants gave informed consent to participate.

**Table 1:** Participant demographics for entire sample (*N* = 456) and for deficient exposure/utopia exposure subsample (*N* = 357).

	***N* (%) *=* 456**	***N* (%) = 357**	**Nature deficient**	**Nature utopia**
**Leaf rating**				
Nature deficient exposure	139 (30.48)	139 (38.94)		
Nature light exposure	16 (3.51)	–		
Nature adequate exposure	26 (5.7)	–		
Nature rich exposure	40 (8.77)	–		
Nature utopia exposure	218 (47.81)	218 (61.06)		
No data	17 (3.73)	–		
	***N*** **(%)** *=* **456**	***N*** **(%)** = **357**	***N*** **(%)** =**139**	***N*** **(%)** =**218**
**Gender**				
Men	224 (49.12)	178 (49.86)	84 (60.43)	94 (43.12)
Women	218 (47.81)	170 (47.62)	51 (36.69)	119 (54.59)
Non-binary, genderfluid, non-conforming, or other	14 (3.01)	9 (2.52)	3 (2.16)	5 (2.29)
**Race and ethnicity** ^a^				
White or European American	292 (64.04)	234 (65.54)	77 (55.40)	157 (72.02)
Black or African American	118 (25.88)	91 (25.49)	42 (30.22)	49 (22.48)
Asian or Asian American	32 (7.02)	24 (6.72)	9 (6.47)	15 (6.88)
Latinx or Hispanic	29 (6.36)	21 (5.88)	12 (8.63)	9 (4.13)
Native American or Alaskan Native	7 (1.54)	4 (1.12)	2 (1.44)	2 (0.92)
Arab or Middle Eastern	4 (0.88)	3 (0.84)	0 (0.00)	3 (1.38)
**SES**				
Working class	166 (36.4)	124 (34.73)	45 (32.37)	79 (36.24)
Lower middle class	161 (35.3)	129 (36.13)	45 (32.37)	84 (38.53)
Upper middle class	125 (27.4)	101 (28.29)	47 (33.81)	54 (24.77)
Upper class	4(0.90)	3 (8.40)	2 (1.44)	1 (0.46)
	***M*** **(*****SD*****)**	***M*** **(*****SD*****)**	***M*** **(*****SD*****)**	***M*** **(*****SD*****)**
Age	39.96 (13.21)	40.04 (13.44)	39.00 (13.58)	40.70 (13.34)
Nature score	60.98 (38.19)	62.08 (41.53)	10.58 (5.46)	94.93 (5.29)

^a^Percentages do not total 100% as participants may have selected more than one answer.

### Materials and measures

#### Affect

We used the 20-item Positive and Negative Affect Scale (PANAS; Watson et al., 1988). Participants respond to 10 adjectives for positive affect (PA) and 10 for negative affect (NA) by rating the extent to which each adjective currently described them on a five-point Likert scale, with higher totals reflecting stronger affect. The PANAS has strong reliability with alpha values of 0.88 for PA and 0.87 for NA. In the current sample, both scales showed good internal consistency, with alpha values of 0.94 (PA pre-test), 0.93 (NA pre-test), 0.95 (PA post-test), and 0.92 (NA post-test). Two additional adjectives, “happy” and “stressed,” were added and scored as separate variables. All 22 items were randomized for both the pre-test and post-test administrations.

#### Digits span backwards (DSB)

Participants completed Digit Span Backward (DSB) tasks from the WAIS-IV (Wechsler, 2008), designed to assess attention and working memory. This task required participants to view 14 digit sequences, with 2 sequences each of lengths from two to eight digits, at a rate of one digit per second and then type those sequences in reverse order (e.g., “3, 2” would be reported as “2, 3”). The total number of correct sequences, out of 14, was calculated. A different set of sequences was administered for the pre-test and the post-test.

#### Perceived restorativeness (PRS)

The Perceived Restorativeness Scale (PRS; Pasini et al., 2014) consists of 11 items on a 7 point Likert scale assessing the restorative quality of an environment. Scores are summed, with higher scores representing more total restorativeness. Reliability in the current sample was good with a Cronbach's alpha of 0.89.

#### Environmental manipulation

Participants were randomly assigned via Qualtrics to one of three environments (Nature, Urban, or Control). To maintain a 45-min procedure and ensure engagement, each condition involved a 5-min video, consistent with prior evidence that 4-min exposures are effective (Meuwese et al., 2021). The nature condition showcased a walk through Plitvice Lakes with accompanying nature sounds (4k Relaxation Channel, 2020). The Urban environment video showed a walk through New York City with accompanying urban sounds (NYC Peaceful Mind, 2023). The Control video displayed various geometric patterns compiled from three different videos (Kuraitou, 2009; Ordinary Folk, 2020, 2024) without sound, as any sounds would be artificial and unrelated to the environment.

#### Nature exposure

Participants' residential zip codes were used to assess their Nature Score and Leaf Rating (Nature Quant Open, 2025). Nature Quant utilizes machine learning to process multiple sources of information, including satellite and other imaging data, pollution measurements, tree canopies, and other factors, to create a Nature Score ranging from 0 to 100, with higher scores representing greater amount and quality of nature (Browning et al., 2024). NatureQuant further divides these scores into 5 Leaf Rating bins, with Nature Scores of 0–19.9 indicating a Nature Deficient environment, 20–39.9 representing a Nature Light environment, 40–59.9 representing Nature Adequate, 60–79.9 indicating Nature Rich, and 80–100 representing Nature Utopia. Thus, Nature Deficient environments are substantially nature-deprived, and few opportunities for nature immersion exist. Nature Light and Nature Adequate environments also exhibit nature deficits, with low to moderate levels of natural elements and opportunities for nature immersion. In contrast, Nature Utopias have high quantity and quality of natural elements with plentiful opportunities for nature immersion.

### Procedure

Participants were recruited through Prolific and redirected to an online Qualtrics survey. After providing informed consent, participants answered pre-test questions assessing their happiness, stress, affect (PANAS), and attention (DSB). Participants were then randomly assigned within Qualtrics to one of the three environmental videos (Nature, Urban, or Control) with instructions to watch attentively. As an attention check, they responded to two prompts afterwards asking them to describe elements from the videos. Next, participants completed post-test measures including happiness, stress, affect (PANAS), attention (DSB), and perceived restorativeness (PRS), followed by various demographic questions (age, gender, race and ethnicity, SES, and residential zip code). At the end of the survey, participants were debriefed, thanked for their participation, and redirected back to Prolific for payment. Participants took an average of 47 min to complete the experiment.

## Results

### Hypothesis 1

We conducted pre-registered analyses to test hypothesis 1 regarding the effect of Environment with a 2 (within subjects: Time: Pre-Test, Post-Test) × 3 (between subjects: Environment: Nature, Urban, Control) repeated-measures ANOVA on each of the outcome variables (“stressed,” “happy,” PA, NA, DSB) for the entire sample (*N* = 456). To test for *post-hoc* (Tukey) differences between the three environmental conditions, we conducted five separate One-Way between-subjects ANOVAs with the factor of Environment (Nature, Urban, Control) on the change score for each of the 5 repeated measures DVs (created by subtracting pre-test scores from post-test scores for “stressed,” “happy,” PA, NA, and DSB). For perceived restorativeness, which was measured only once at post-test, we conducted a One-Way between subjects ANOVA with the factor of Environment on the post-test score for perceived restorativeness. The means and standard deviations for the pre-test and post-test scores of each of these variables as a function of Time and Environment can be found in Table 2 and the ANOVA summary statistics (*F, df, p*, and η_*p*_^2^) can be found in Table 3.

**Table 2:** Descriptive statistics for outcome measures as a function of environment in the full sample (*N* = 456).

**Positive affect**	**Pre-test**		**Post-test**		**Change score**	***N***
	***M***	***SD***	***M***	***SD***	***M*** **(*****SD*****)**	
Nature environment	33.39	10.32	34.17	10.53	0.79 (4.29)	187
Urban environment	32.09	10.65	31.87	10.94	−0.22 (5.75)	183
Control environment	32.88	10.58	33.01	11.65	0.13 (5.47)	86
**Happiness**	**Pre-test** ^1^		**Post-test** ^1^		**Change score**	***N***
	***M***	***SD***	***M***	***SD***	***M*** **(*****SD*****)**	
Nature environment	3.34	1.20	3.51	1.20	0.17 (0.92)	187
Urban environment	3.21	1.29	3.21	1.35	−0.01 (0.99)	183
Control environment	3.20	1.33	3.34	1.33	0.14 (1.01)	86
**Negative affect** ^*^	**Pre-test** ^1^		**Post-test** ^1^		**Change score**	***N***
	***M***	***SD***	***M***	***SD***	***M*** **(*****SD*****)**	
Nature environment^a^	14.96	7.09	13.95	6.33	−1.01 (3.38)	187
Urban environment^a, b^	14.42	6.54	14.27	6.53	−0.15 (3.50)	183
Control environment^b^	14.31	5.89	13.06	4.60	−1.26 (3.90)	86
**Stress** ^*^	**Pre-test** ^1^		**Post-test** ^1^		**Change score**	***N***
	***M***	***SD***	***M***	***SD***	***M*** **(*****SD*****)**	
Nature environment	2.02	1.18	1.76	1.10	−0.25 (0.81)	187
Urban environment	1.89	1.07	1.82	1.06	−0.07 (0.91)	183
Control environment	1.95	1.17	1.63	0.95	−0.33 (1.01)	86
**Digit span backwards**	**Pre-test** ^1^		**Post-test** ^1^		**Change score**	***N***
	***M***	***SD***	***M***	***SD***	***M*** **(*****SD*****)**	
Nature environment	9.21	2.82	9.96	2.79	0.75 (2.75)	187
Urban environment	9.07	2.83	9.72	3.03	0.66 (2.09)	183
Control environment	9.26	2.92	9.28	3.17	0.02 (2.22)	86
**Restorativeness** ^2^			***M***	***SD***		***N***
Nature environment^3, 4^			61.56	9.22		187
Urban environment^3^			52.30	13.01		183
Control environment^4^			52.15	12.24		86

The numerical superscript ^1^ indicates a main effect of Time for that outcome measure, with significant differences (*p* < 0.05) between Pre-test and Post-test scores. Numerical superscript ^2^ indicates a significant main effect for Environment (nature, urban, control), and superscripts ^3^ and ^4^ indicate significant post-hoc (Tukey) differences (*p* < 0.05) between environments with the same superscript. Perceived restorativeness was only measured once, with Post-test measures.

^*^ Indicates a significant interaction between Time and Environment, with each alphabetical superscript ^a, b^ indicating a significant post-hoc (Tukey) difference (*p* < 0.05) between groups with the same superscript for that outcome measure.

**Table 3:** ANOVA summary statistics for the effects of environment on outcome measures in the full sample (*N* = 456).

	***F***	***df***	** $\eta_{p}^{2}$ **	***p***
**Positive affect (PA)**				
Time	0.81	1, 453	0.00	0.37
Environment	1.39	2, 453	0.01	0.25
Time × environment	1.80	2, 453	0.01	0.17
**Happiness**				
Time^*^	4.46	1, 453	0.01	0.04
Environment	1.56	2, 453	0.01	0.21
Time × environment	1.67	2, 453	0.01	0.19
**Negative affect (NA)**				
Time^**^	20.85	1, 453	0.04	< 0.001
Environment	0.48	2, 453	0.00	0.62
Time × environment^*^	4.03	2, 453	0.02	0.02
**Stress**				
Time^**^	25.53	1, 453	0.05	< 0.001
Environment	0.29	2, 453	0.00	0.75
Time × environment^*^	3.06	2, 453	0.01	0.048
**Attention (DSB)**				
Time^**^	15.85	1, 453	0.03	< 0.001
Environment	0.49	2, 453	0.00	0.61
Time × environment	2.89	2, 453	0.01	0.06
**Perceived restorativeness**				
Environment^**^	36.48	2, 453	0.14	< 0.001

^*^p < 0.05; ^**^p < 0.01.

#### Positive affect

There was no significant main effect of Time or Environment and no interaction between Time and Environment. Thus, *post-hoc* tests are not reported.

#### Happiness

There was a significant small main effect of Time, such that happiness increased from pre-test to post-test. There was no main effect of Environment and no significant interaction between Time and Environment. Thus, *post-hoc* tests are not reported.

#### Negative affect

There was a significant small main effect of Time, such that negative affect decreased from pre-test to post-test. There was no main effect of Environment. There was a significant small interaction between Time and Environment. *Post-hoc* (Tukey) tests on the change score from pre-test to post-test revealed that those in both the Nature (*M* = −1.01, *SD* = 3.38, *p* = 0.05) and the Control (*M* = −1.26, *SD* = 3.90, *p* = 0.04) environments experience a larger decrease in negative affect than those in the Urban environment (*M* = −0.15, *SD* = 3.50), and the Nature and Control environments did not differ from each other (*p* = 0.86).

#### Stress

There was a significant small main effect of Time, such that stress decreased from pre-test to post-test. There was no main effect of Environment. There was a small significant interaction between Time and Environment. *Post-hoc* (Tukey) tests on the change score from pre-test to post-test revealed no significant differences between the Nature (*M* = −0.25, *SD* = 0.81) and either the Control (*M* = −0.33, *SD* = 1.01, *p* = 0.80) or the Urban (*M* = −0.07, *SD* = 0.91, *p* = 0.13), and the difference between Control and Urban approached significance (*p* = 0.08).

#### Attention

There was a significant small main effect of Time, such that performance on the DSB increased from pre-test to post-test. There was no main effect of Environment and no significant interaction between Time and Environment. Thus, *post-hoc* tests are not reported.

#### Perceived restorativeness

There was a significant large effect of Environment. *Post-hoc* (Tukey) tests revealed that the Nature (*M* = 61.56, *SD* = 9.22) environment was significantly more restorative than the Control (*M* = 52.15, *SD* = 12.24, *p* < 0.001) and Urban (*M* = 52.30, *SD* = 13.01, *p* < 0.001) environments, which did not differ from each other (*p* = 0.995).

### Hypothesis 2

To test hypothesis 2, that Exposure would moderate the relationship between Environment and the outcome measures, we conducted pre-registered 2 (within-subjects: Time: Pre-Test, Post-Test) × 3 (between-subjects: Environment: Nature, Urban, Control) × 2 (between-subjects: Exposure: Nature Deficient, Nature Utopia) repeated-measures ANOVAs on each of the outcome variables (“stressed,” “happy,” PA, NA, DSB) for the subsample (*N* = 357). Additionally, a 3 (Environment: Nature, Urban, Control) × 2 (Exposure: Nature Deficient, Nature Utopia) between-subjects ANOVA was conducted on perceived restorativeness. To test for *post-hoc* differences between all six conditions, we conducted a One-Way between-subjects ANOVA with the factor of Environment/Exposure Condition (Nature Environment/Deficient Exposure; Nature Environment/Utopia Exposure; Control Environment/Deficient Exposure; Control Environment/Utopia Exposure; Urban Environment/Deficient Exposure; Urban Environment/Utopia Exposure) on the change score (post-test minus pre-test) for “stressed,” “happy,” PA, NA, and DSB, and the post-test score for perceived restorativeness (which was only measured once at post-test). The means and standard deviations for the pre-test and post-test scores of each of these variables as a function of Time, Environment, and Exposure can be found in Table 4, and the ANOVA summary statistics can be found in Table 5. As these analyzes were performed on a subsample of the full sample analyzed for hypothesis 1, all main effects and interactions are reported below.

**Table 4:** Descriptive statistics for outcome measures as a function of environment and exposure in those with nature deficient and nature utopia exposure (*N* = 357).

**Positive affect**	**Pre-test**		**Post-test**		**Change score**	***N***
	***M***	***SD***	***M***	***SD***	***M*** **(*****SD*****)**	
Nature environment	34.09	10.01	34.74	10.40	0.65 (3.92)	149
Deficient exposure	38.79	8.79	39.51	8.82	0.66 (0.64)	63
Utopia exposure	30.64	9.46	31.24	10.12	0.65 (0.55)	86
Urban environment	31.95	10.89	31.72	10.83	−0.24 (5.80)	144
Deficient exposure	36.10	10.61	36.80	9.82	0.71 (0.71)	51
Utopia exposure	29.68	10.40	28.92	10.37	−0.75 (0.53)	93
Control environment	33.14	10.56	32.66	11.36	−0.48 (5.76)	64
Deficient exposure	35.80	13.03	36.24	12.90	0.44 (1.02)	25
Utopia exposure	31.44	8.37	30.36	9.75	−1.08 (0.81)	39
Deficient exposure total^5^	37.27	10.34	37.93	10.04	0.66 (4.42)	139
Utopia exposure total^5^	30.37	9.68	30.10	10.17	−0.28 (5.47)	218
**Happiness**	**Pre-test** ^1^		**Post-test** ^1^		**Change score**	***N***
	***M***	***SD***	***SD***	***SD***	***M*** **(*****SD*****)**	
Nature environment	3.40	1.21	3.58	1.18	0.18 (0.94)	149
Deficient exposure	3.73	1.15	4.00	1.03	0.28 (0.92)	63
Utopia exposure	3.15	1.19	3.57	1.19	0.11 (0.96)	86
Urban environment	3.19	1.35	3.16	1.35	−0.03 (1.00)	144
Deficient exposure	3.59	1.42	3.76	1.21	0.18 (0.93)	51
Utopia exposure	2.98	1.26	2.93	1.32	−0.15 (1.02)	93
Control environment	3.20	1.30	3.34	1.28	0.14 (0.99)	64
Deficient exposure	3.52	1.53	3.72	1.37	0.20 (1.12)	25
Utopia exposure	3.00	1.10	3.10	1.17	0.10 (0.91)	39
Deficient exposure total^5^	3.64	1.32	3.86	1.16	0.22 (0.96)	139
Utopia exposure total^5^	3.05	1.20	3.05	1.25	0.00 (0.98)	218
Negative affect^*^~	**Pre-test** ^1^		**Post-test** ^1^		**Change score**	***N***
	***M***	***SD***	***M***	***SD***	***M*** **(*****SD*****)**	
Nature environment	14.23	6.89	13.49	5.90	−0.75 (2.99)	149
Deficient exposure	14.46	6.58	13.73	6.51	−0.75 (2.65)	63
Utopia exposure	14.07	6.80	13.31	5.45	−0.74 (3.23)	86
Urban environment^a^	13.70	5.19	13.75	5.79	0.05 (3.40)	144
Deficient exposure^g^	14.27	5.89	14.49	6.90	0.22 (2.98)	51
Utopia exposure^h^	13.39	4.78	13.34	5.07	−0.04 (3.62)	93
Control environment^a^	14.37	5.78	12.95	4.08	−1.42 (4.26)	64
Deficient exposure	12.56	4.15	12.16	3.53	−0.40 (2.75)	25
Utopia exposure^g, h^	15.54	6.39	13.46	4.37	−2.08 (4.92)	39
Deficient exposure total	14.05	5.95	13.73	6.25	−0.32 (2.81)	139
Utopia exposure total	14.04	5.96	13.35	5.09	−0.69 (3.79)	218
**Stress**	**Pre-test** ^1^		**Post-test** ^1^		**Change score**	***N***
	***M***	***SD***	***M***	***SD***	***M*** **(*****SD*****)**	
Nature environment	1.92	1.12	1.68	1.00	−0.23 (0.77)	149
Utopia exposure	1.97	1.20	1.76	1.05	−0.19 (0.84)	86
Urban environment	1.89	1.09	1.81	1.10	−0.08 (0.95)	144
Deficient exposure	1.67	0.93	1.76	1.16	0.10 (0.88)	51
Utopia exposure	2.01	1.16	1.84	1.07	−0.017 (0.97)	93
Control environment	1.97	1.20	1.63	0.86	1.01 (0.13)	64
Deficient exposure	1.64	0.95	1.40	0.71	−0.24 (1.13)	25
Utopia exposure	2.18	1.30	1.77	0.93	−0.41 (0.94)	39
Deficient exposure total^5^	1.75	0.96	1.62	0.99	−0.13 (0.85)	139
Utopia exposure total^5^	2.02	1.20	1.79	1.03	−0.23 (0.92)	218
**Digit span backwards**	**Pre-test** ^1^		**Post-test** ^1^		**Change score**	***N***
	***M***	***SD***	***M***	***SD***	***M*** **(*****SD*****)**	
Nature environment	9.23	2.76	9.99	2.76	0.75 (2.93)	149
Deficient exposure	9.11	3.17	10.22	2.98	1.09 (3.25)	63
Utopia exposure	9.33	2.44	9.81	2.59	0.49 (2.65)	86
Urban environment	8.99	2.89	9.70	3.15	0.71 (2.02)	144
Deficient exposure	9.20	2.88	10.22	3.44	1.02 (2.30)	51
Utopia exposure	8.88	2.90	9.42	2.96	0.54 (1.84)	93
Control environment	9.22	2.89	9.31	3.24	0.09 (2.16)	64
Deficient exposure	9.92	2.41	9.92	2.90	0.00 (1.66)	25
Utopia exposure	8.77	3.11	8.92	3.42	0.15 (2.44)	39
Deficient exposure total	9.29	2.94	10.17	3.12	0.88 (2.72)	139
Utopia exposure total	9.04	2.76	9.49	2.92	0.45 (2.29)	218
**Restorativeness** ^2^			***M***	***SD***		***N***
Nature environment^3, 4^			61.34	9.46		149
Deficient exposure^b, c, d^			62.44	9.17		63
Utopia exposure^e, f^,			60.52	9.64		86
Urban environment^3^			52.71	13.00		144
Deficient exposure^b^			55.10	13.14		51
Utopia exposure^d, e^			51.40	12.80		93
Control environment^4^			52.88	12.38		64
Deficient exposure			56.12	13.69		25
Utopia exposure^c, f^			50.79	11.15		39
Deficient exposure total^5^			58.61	12.06		139
Utopia exposure total^5^			54.89	12.18		218

The numerical superscript ^1^ indicates a main effect of Time for that outcome measure, with significant differences (*p* < 0.05) between Pre-test and Post-test scores. Numerical superscript ^2^ indicates a significant main effect for Environment (nature, urban, control), and superscripts ^3^ and ^4^ indicate significant post-hoc (Tukey) differences (*p* < 0.05) between environments with the same superscript. Perceived restorativeness was only measured once, with Post-test measures. Numerical superscript ^5^ indicates a significant main effect for Exposure for that outcome measure, with significant differences (*p* < 0.05) between Deficient Exposure and Utopia Exposure.

^*^ Indicates a significant interaction between Time and Environment, with the alphabetical superscript ^a^ indicating a significant post-hoc (Tukey) difference (*p* < 0.05) between groups with the same superscript for that outcome measure.

# Indicates a significant interaction between Environment and Exposure, with each alphabetical superscript ^b, c, d, e, f^ indicating a significant post-hoc (Tukey) difference (*p* < 0.05) between groups with the same superscript for that outcome measure.

~ Indicates a significant interaction between Time, Environment, and Exposure, with each alphabetical superscript ^g, h^ indicating a significant post-hoc (Tukey) difference (*p* < 0.05) between groups with the same superscript for that outcome measure.

**Table 5:** ANOVA summary statistics for the effects of environment and chronic nature exposure on outcome measures in those with nature deficient and nature utopia exposure (*N* = 357).

	***F***	***df***	** $\eta_{p}^{2}$ **	***p***
**Positive affect (PA)**				
Time	0.13	1, 351	0.00	0.72
Environment	1.39	2, 351	0.01	0.17
Exposure^**^	36.12	1, 351	0.09	< 0.001
Time × Environment	1.04	2, 351	0.01	0.36
Time × Environment × Exposure	0.75	2, 351	0.00	0.47
**Happiness**				
Time^*^	4.37	1, 351	0.01	0.04
Environment	1.78	2, 351	0.01	0.17
Exposure^**^	25.15	1, 351	0.07	< 0.001
Time × Environment	1.24	2, 351	0.01	0.29
Time × Environment × Exposure	0.40	2, 351	0.00	0.67
**Negative affect (NA)**				
Time^**^	10.04	1, 351	0.03	0.002
Environment	0.17	2, 351	0.00	0.84
Exposure	0.14	1, 351	0.00	0.17
Time × Environment^*^	3.76	2, 351	0.02	0.02
Time × Environment × Exposure	1.32	2, 351	0.01	0.27
**Stress**				
Time^**^	14.78	1, 351	0.04	< 0.001
Environment	0.12	2, 351	0.00	0.89
Exposure^*^	5.53	1, 351	0.02	0.02
Time × Environment	2.83	2, 351	0.02	0.06
Time × Environment × Exposure	1.23	2, 351	0.01	0.29
**Attention (DSB)**				
Time^**^	14.66	1, 351	0.04	< 0.001
Environment	0.25	2, 351	0.00	0.78
Exposure	3.44	1, 351	0.01	0.46
Time × Environment	2.10	2, 351	0.01	0.12
Time × Environment × Exposure	0.55	2, 351	0.00	0.58
**Perceived restorativeness**				
Environment^***^	21.09	2, 351	0.11	< 0.001
Exposure^**^	7.40	1, 351	0.02	0.007
Environment × Exposure	0.52	2, 351	0.00	0.60

^*^p < 0.05; ^**^p < 0.01.

#### Positive affect (PA)

As in the full sample, there was no significant main effect of Time or Environment. There was a significant medium effect of Exposure, such that those who resided in Nature Deficient areas had higher PA than those who resided in Nature Utopias. There was no interaction between Time and Environment, and Exposure did not moderate a relationship between Time and Environment. Thus, *post-hoc* tests are not reported.

#### Happiness

As in the full sample, there was again a significant small main effect of Time, such that happiness increased from pre-test to post-test. There was no significant main effect of Environment. There was a significant medium effect of Exposure, such that those who resided in Nature Deficient areas had higher happiness ratings than those who resided in Nature Utopias. There was no interaction between Time and Environment, and Exposure did not moderate the relationship between Time and Environment. Thus, *post-hoc* tests are not reported.

#### Negative affect (NA)

As in the full sample, there was again a significant small main effect of Time, such that NA decreased from pre-test to post-test. There were no significant main effects of Environment or Exposure. There was a significant small interaction between Time and Environment, but Exposure did not moderate the relationship between Time and Environment. *Post-hoc* (Tukey) tests on the change score from pre-test to post-test revealed that those in the Control environment experienced a larger NA decrease than those in the Urban environment (*p* = 0.01), but the Nature environment did not differ from either the Control or Urban (*p*s > 0.05). As exposure did not moderate the relationship between Time and Environment, further *post-hoc* tests are not reported.

#### Stress

As in the full sample, there was again a significant small main effect of Time, such that stress decreased from pre-test to post-test. There was no significant main effect of Environment. There was a significant small effect of Exposure, such that those residing in Nature Utopias reported more stress than those residing in Nature Deficient environments. Contrary to the full sample, the interaction between Time and Environment was not significant. Finally, exposure did not moderate the relationship between Time and Environment. Thus, *post-hoc* tests are not reported.

#### Attention (DSB)

As in the full sample, there was again a significant small main effect of Time, such that DSB performance increased from pre-test to post-test. There was no significant main effect of Environment and no significant effect of Exposure. Further, there was no interaction between Time and Environment, and Exposure did not moderate a relationship between Time and Environment. Thus, *post-hoc* tests are not reported.

#### Perceived restorativeness

Similar to the full sample, there was a significant medium main effect of Environment. There was also a significant small effect of Exposure, such that those residing in Nature Deficient areas perceived environments as more restorative than those residing in Nature Utopias. Finally, exposure did not moderate the effect of Environment on restorativeness.

## Discussion

The present study investigated whether chronic exposure to nature moderated the effects of acute nature exposure on affect and attention. Regardless of environments, outcomes (except PA) improved consistently from pre- to post-test, likely reflecting practice effects for DSB and video enjoyment. Partially supporting our first hypothesis, the Time × Environment interaction was significant for NA (both samples) and stress (full sample), but not for PA, happiness, or DSB. Interestingly, while both the Nature and Control conditions showed greater reduction in NA than the Urban condition in the full sample, only the Control condition resulted in a larger NA decrease than the Urban condition in the subsample. Nature and Urban did not differ from each other for PA, happiness, stress, or DSB. Further, Nature and Control did not differ from each other on PA, happiness, NA, stress, or DSB, suggesting that the effects of the Nature and Control condition on affect and attention were similar.

A strength of our pre-test/post-test design is that it allowed us to test both ART and N-A-R. While only the Nature video contained nature content, both the Nature and Control videos were likely positively valenced, which may explain reductions in negative affect for those conditions. The geometric control in our study was chosen because it contained no natural or urban elements, yet geometric patterns of varying complexity are pleasant (Bies et al., 2016; Gómez-Puerto et al., 2016; Lavdas and Schirpke, 2020; Redies et al., 2007). Indeed, (Joye et al. 2024b) found that viewing beautiful fractal patterns enhanced effort and motivation in ticking tasks, with pleasantness directly predicting these outcomes. The findings of no significant differences in this study on affect or attention measures between the Control and the Nature conditions, both containing pleasant stimuli but differing in nature exposure, support N-A-R (Joye et al., 2024b,a). While our study showed the predicted medium to large effects of Environment on perceived restorativeness, with the Nature condition having the largest restorativeness, and the Control and Urban not different from each other, this difference in perceived restorativeness did not coincide with a difference in attention or affect measures, contrary to ART. Likewise, these results do not support SRT, as there were no differences between the Nature and Urban conditions on stress in either sample.

Thus, these results only offer limited support for the benefits of nature compared to urban exposure and add to the inconsistent findings in this area. These results suggest that while acute nature exposure can reduce negative affect, it does not necessarily improve positive affect, contradicting previous studies reporting improvement in both (Berman et al., 2012; Bratman et al., 2015; Sato and Conner, 2013). Further, these results suggest that exposure to other stimuli (e.g., geometric shapes) may have effects similar to exposure to nature.

Our second hypothesis was that chronic (i.e., residential) exposure to nature would moderate the effects of environment on affect and cognition, with larger effects of environment for those with minimal (e.g., Nature Deficient) prior exposure than those with considerable (e.g., Nature Utopia) prior exposure. The results were again non-supportive, with no moderating effect of exposure on happiness, PA, NA, stress, attention, or perceived restorativeness. Thus, findings suggest that adaptation may not explain inconsistent effects of nature exposure on affect and cognition (Trammell and Aguilar, 2021; Trammell et al., 2024). Encouragingly, it is evident that chronic exposure to nature does not diminish the benefits of acute nature exposure.

Interestingly, while we did not hypothesize a main effect for prior exposure, there were significant differences between those residing in Nature Utopia and Nature Deficient areas in overall happiness, PA, stress, and perceived restorativeness. Surprisingly, those in the Nature Deficient environments consistently showed greater wellbeing (e.g., higher PA and happiness, lower stress) than those in Nature Utopias, contrasting prior work that links greener residential areas to higher wellbeing (Alcock et al., 2014; Beyer et al., 2014; Garrett et al., 2021; Li et al., 2018; Thompson et al., 2012). As SES was not significantly different for those in Deficient or Utopia environments, it cannot explain these results. Participants from Nature-Deficient and Nature-Rich areas in this online study may differ in other factors that are relevant to wellbeing; however, these difference(s) require further investigation.

While this work does not support the hypothesis that chronic exposure weakens the effects of acute nature exposure on affect and attention, limitations exist that warrant investigation. First, our initial data collection was strongly biased toward those residing in Nature Utopia environments. We then collected additional data, recruiting those in Nature Deficient environments. However, we were only able to achieve 30% of our sample as Nature Deficient, and less than 20% in a combined midrange (Nature Light, Nature Adequate, and Nature Rich). This resulted in a bimodal distribution, and we could not examine midrange nature exposure or determine if a curvilinear relationship exists.

Second, due to the need to recruit more participants who lived in Nature Deficient environments, the majority of Nature Utopia data was collected toward the beginning of our data collection (e.g., during the Fall of 2024) and the majority of the Nature Deficient data collected toward the end of data collection (Winter 2024/2025). It is possible that seasonality can account for some of the affective differences observed between Nature Utopia and Nature Deficient participants. However, past research has shown that affective measures, such as mood (Winthorst et al., 2020) and depressive symptoms (Geoffroy et al., 2014; Goldschmied et al., 2025; Harmatz et al., 2000; Øverland et al., 2019) typically worsen in winter. Our findings of higher positive affect and happiness and lower stress for Nature Deficient participants, whose data was mostly collected in winter, suggests that seasonality does not explain these differences. Nevertheless, future studies may consider investigating how seasonality interacts with nature exposure.

Third, while we utilized residential zip code to obtain a comprehensive measure of nature quality and quantity (NatureQuant) as a measure of chronic nature exposure, it should be noted that residing in a Nature Deficient or Nature Utopia environment is not a perfect proxy for actual nature exposure. For example, those in Nature Deficient environments may travel to nature-rich environments for work or pleasure, and those in Nature Utopia environments may remain indoors much of the time. Likewise, nearby quantity and quality of nature do not necessarily correlate with a lifetime of exposure; i.e., some participants could have resided in their zip code for most or, conversely, for very little of their recent lives. Despite these limitations, location information technologies similar to Nature Quant have been used in many prior studies to create various estimates of nature exposure (such as tree cover or “greenness”) that correlate with measures of affect and cognition (Cox et al., 2017; Li et al., 2018; Shanahan et al., 2016). Future research should include additional measures of chronic exposure, such as actual exposure across various environments and a more detailed residential history.

Finally, while short nature exposure under 10 min are effective (e.g., Meuwese et al., 2021; Palanica et al., 2019; Van den Berg et al., 2003), longer durations of 30 min (Bell et al., 2025) may have resulted in larger effects. Additionally, due to time constraints, we only included one measure of attention, DSB, which showed no effects of or interactions with the environment. While DSB has been shown to have larger effects than many other cognitive tasks (Bell et al., 2025), additional measures of attention and working memory may have revealed other effects.

Future research may benefit from a stronger (i.e., in-person) and longer environmental manipulation. Since recruiting locally may limit variation in chronic nature exposure, studies could test visitors or tourists in natural areas or sample across regions while controlling for demographics. Online studies have a higher potential for a wide range of nature exposure, although recruitment should carefully ensure a diverse range. Future research should also explore the surprising effects of Nature Deficient participants having higher happiness, PA, and lower stress than Nature Utopia participants.

## Conclusions

In conclusion, effects of the environment on affect and attention, as with past studies, remain inconsistent. Chronic exposure does not moderate the relationship between acute environmental exposure and affect or attention in this sample, although future research with larger samples and longer acute exposure is needed. Previously published research generally examines only acute exposure (without taking into account chronic exposure) or chronic exposure (without looking at an acute exposure). We urge additional explorations, as this is the first study to our knowledge to examine the interplay of chronic and acute exposure. As the concept of spending time in nature to improve wellbeing has become popularized, the finding that those who reside in either Nature Deficient or Nature Utopia environments respond similarly to acute nature exposure implies that the effects of nature can be universal and do not depend on one's history of nature exposure.

## Data Availability

The raw data supporting the conclusions of this article will be made available by the authors, without undue reservation.
